# Collaborative Biomedicine in the Age of Big Data: The Case of Cancer

**DOI:** 10.2196/jmir.2496

**Published:** 2014-04-07

**Authors:** Abdul R Shaikh, Atul J Butte, Sheri D Schully, William S Dalton, Muin J Khoury, Bradford W Hesse

**Affiliations:** ^1^PricewaterhouseCoopers LLPMcLean, VAUnited States; ^2^Division of Systems MedicineDepartment of PediatricsStanford University School of MedicineStanford, CAUnited States; ^3^Division of Cancer Control and Population SciencesNational Cancer InstituteBethesda, MDUnited States; ^4^DeBartolo Family Personalized Medicine Institute at the Moffitt Cancer CenterMoffitt Cancer CenterTampa, FLUnited States

**Keywords:** biomedical research, crowdsourcing, health information technology, innovation, precision medicine

## Abstract

Biomedicine is undergoing a revolution driven by high throughput and connective computing that is transforming medical research and practice. Using oncology as an example, the speed and capacity of genomic sequencing technologies is advancing the utility of individual genetic profiles for anticipating risk and targeting therapeutics. The goal is to enable an era of “P4” medicine that will become increasingly more predictive, personalized, preemptive, and participative over time. This vision hinges on leveraging potentially innovative and disruptive technologies in medicine to accelerate discovery and to reorient clinical practice for patient-centered care. Based on a panel discussion at the Medicine 2.0 conference in Boston with representatives from the National Cancer Institute, Moffitt Cancer Center, and Stanford University School of Medicine, this paper explores how emerging sociotechnical frameworks, informatics platforms, and health-related policy can be used to encourage data liquidity and innovation. This builds on the Institute of Medicine’s vision for a “rapid learning health care system” to enable an open source, population-based approach to cancer prevention and control.

## Introduction

Biomedicine is undergoing a revolution driven by innovation in high throughput and connective computing [1,2], big data [3,4], and evolving models of individual and population care [5,6]. Emerging informatics technologies and platforms are being used to combine molecular, clinical, and population data to better anticipate risk, target therapeutics, and manage care for cancer and other diseases [6,7]. Based on a panel discussion at the 2012 Medicine 2.0 conference (5th World Congress on Social Media, Mobile Apps, and Internet/Web 2.0 in Health and Medicine) at the Harvard Medical School conference center in Boston with representatives from the National Cancer Institute (NCI), Moffitt Cancer Center (MCC), and Stanford University School of Medicine, this paper explores how emerging technologies and innovative care models that build on the concepts of “P4” medicine (ie, predictive, personalized, preemptive, and participatory) [8] and the learning health care system [9] can help enable an open source, population-based approach to cancer prevention and control.

## P4 Medicine and the Learning Health System

The growing speed and capacity of genomic sequencing technologies are advancing the utility of individual genetic profiles for anticipating risk and targeting therapeutics for cancer [10]. Combining the digital revolution with genomics and other “omics” fields, the term P4 medicine implies a systems approach to biology and medicine that brings together molecular immunology, advanced computation, biotechnology, and genomics, among other fields [8]. Standing for *predictive* profiles of risk*, preventive* clinical and wellness systems, *personalized* medicine*,* and *participative* research and practice*,* proponents of P4 medicine extend its purview beyond genomics to include multiple data vectors such as longitudinal molecular, cellular, and phenotypic data for predicting disease progression and targeting intervention [11]. Thus, P4 medicine is predicated on the notion that individual disease and broader notions of health and wellness can be quantified with advanced computation and informatics through systems approaches to decipher the inherent complexity of billions of data points surrounding patients in the future.

P4 medicine can be viewed in conjunction with the Institute of Medicine’s (IOM) concept of a learning health system, which simultaneously links effective, efficient clinical health care to the biomedical research enterprise [12]. As presented in a draft proposal at the IOM National Cancer Policy Forum, the notion of a rapid learning health system for cancer utilizes basic translational, comparative effectiveness, and health services research synchronized with optimal delivery of precision care. This model of research and practice is based on two elements: (1) a sufficiently advanced digital health infrastructure that can fully utilize the phenomenon of (2) data liquidity, defined as “the rapid, seamless, secure exchange of useful, standards-based information among authorized individual and institutional senders and recipients” [13].

Both of these innovative approaches to health research and practice—P4 medicine and the learning health system—require robust technology infrastructure and data liquidity to realize the ambitious aim of transforming biomedicine for cancer and other diseases. Moreover, in presenting the rapid learning health system framework for cancer research and practice, members of the National Cancer Policy Forum’s planning committee identified five challenges to developing a learning health system for cancer that are also directly relevant to the realization of P4 medicine: (1) data collection (eg, data accuracy, timeliness, and completeness), (2) incentivizing data-sharing, (3) data standards, harmonization, and computation, (4) meaningful use of health IT, and (5) the central role of government entities such as the National Institutes of Health (NIH), the Food and Drug Administration, and the Centers for Medicare and Medicaid Services [13].

One example of a rapid learning health care system that is currently being implemented for oncology is the American Society of Clinical Oncology’s Cancer Learning Intelligence Network for Quality (CancerLinQ) system [14]. CancerLinQ is designed to address the growing challenge of managing the deluge of data emerging from precision medicine for cancer care. The system incorporates data from researchers, providers, and patients in order to continually improve comprehensive clinical algorithms reflecting preferred care at a series of decision nodes for clinical decision support.

## “P5 Medicine”: A Population Approach to Transforming Biomedicine

Adding both promise and complexity to the previously described frameworks of modern care, proponents of the public health sciences assert that a 5^th^ P standing for a *population* perspective is needed to realize the full potential of P4 medicine [15]. Limited by a primary focus on individual health, the P4 approach to biomedicine can be augmented as follows:

- Predictive: Predicting health using systems biologic and phenotypic information augmented with the ecological model of health to account for multilevel determinants of health and life-course approaches.
- Preventive: Early disease detection and prevention also incorporate population screening principles to assess benefits, harm, and costs of primary prevention.
- Personalized: Targeted therapeutics and diagnostics enhanced by principles of evidence-based medicine using formal analytic frameworks for comparative effectiveness.
- Participatory: Engaging patients, providers, and systems including the public health enterprise (eg, policy development, regulatory science, implementation, and health services research).

In addition to the rapid learning health system approach, which incorporates notions of translational, comparative effectiveness and health services research, adding a population focus to P4 medicine explicitly addresses broader, structural issues such as costs and potential for harm that result in greater social, economic, and health disparities. Population science also helps focus on the need for enhanced population level interventions (such as education, employment, and roads) in addition to individual level interventions to improve health and prevent disease [15].

One innovative example of an effort incorporating a P5 approach to cancer biomedicine can be found in the Moffitt Cancer Center’s Total Cancer Care (TCC) proposal for a new federated model for research and health care [16]. Based on a robust informatics platform allowing for real-time integration and analysis of disparate multilevel data, the TCC builds on the rapid learning health system model by incorporating development of “secondary use” of data including comparative effectiveness research. Perhaps equally important, TCC proposes a shared governance approach with a federated data model designed to promote team science, data liquidity, and access to the disparate data sources that are essential for effective transformation of the biomedical enterprise [17].

## Crowdsourcing Science and the Future of Biomedicine

Scaling the biomedical research enterprise to tackle cancer and other diseases with unknown therapeutics and unclear diagnostics will require recruiting new communities of investigators such as those in engineering and computational disciplines, often earlier in their careers. In addition to exploring new models of cancer research and practice, the Medicine 2.0 conference panel also delved into how big data, emerging technologies, and commoditized access to sophisticated wet lab tools and computational methods can spark scientific innovation in basic and applied research. Individuals have greater access to potentially disruptive technologies in medicine to accelerate basic discovery science and reorient clinical practice for patient-centered care. Publically available molecular measurements can be used to discover novel biomarkers of disease [18] and can be used to find novel uses for existing therapeutics [19,20]. One example of individuals addressing big data challenges is in the field of computational immunogenics, where a challenge sponsored by Harvard Medical School was used to crowdsource solutions that significantly outperformed leading academic efforts [21]. Such immunology datasets, including clinical trials, are available at the National Institute of Allergy and Infectious Diseases ImmPort website for professionals and students.

On a policy level, examples such as the federal Open Government Initiative, the America COMPETES Act, and NIH requirements for data sharing in grant proposals, combined with public and private sector initiatives by donors, journals, and foundations, have led to unprecedented amounts of data being available for secondary research. Two examples include the Data.gov platform, which enables public access to “nearly 450,000 datasets…across 172 federal agencies” [22], and the availability of one million gene expression microarray measurements for research [23].

In addition to greater availability of data, public and private entities are leveraging prize and challenge mechanisms to accelerate innovation with health-related data. Biomedically related open innovation challenges such as these often involve the release of data first, with the expectation that the “winner” of the challenge is awarded a prize. This is the reverse of the typical grant funding mechanism, with money given first, potentially followed by results [24]. In the cancer arena, NCI and the Office of the National Coordinator for Health Information Technology (ONC) have had success in combining open innovation challenges with the federal Small Business Innovation Research (SBIR) grant program to support the evaluation and dissemination of evidence-based applications for cancer prevention and control [25,26]. For these agencies, the federal prize and challenge mechanism has provided a high-value approach to addressing their core agency missions through building a new ecosystem of developers, entrepreneurs, and scientists who can innovate for cancer control and public health. The most recent ONC and NCI challenge competition, focusing on technology innovation for cancer survivors, expands the Department of Health and Human Services innovation portfolio by incorporating crowdfunding to potentially enhance engagement and market validation of submitted innovations with consumer audiences [27].

## Conclusions

In many respects, cancer is the prototypical workspace for applying new models of scientific discovery and medical practice. The story of cancer is a story of how the body’s complex coding systems go awry through the creation of self-perpetuating errors in cellular replication and growth. Fortunately, advances in genetic sequencing technologies, high throughput data architectures, massively networked public and scientific communities, and the wide availability of sophisticated wet lab tools may be sparking the innovation in “open source” science needed to accelerate progress against the disease. As one panel member put it, “individuals in garages and dorm rooms have greater access to potentially disruptive technologies in medicine than the most well-resourced scientists of the last decade.” This exciting era of distributed and open source science holds great potential for accelerating basic discovery and reorienting clinical practice for patient-centered care and population health.

## Abbreviations

CancerLinQ: Cancer Learning Intelligence Network for Quality
IOM: Institute of Medicine
MCC: Moffitt Cancer Center
NCI: National Cancer Institute
NIH: National Institutes of Health
ONC: Office of the National Coordinator for Health Information Technology
P4: predictive, personalized, preemptive, participatory
SBIR: Small Business Innovation Research
TCC: Total Cancer Care
